# Neural Oscillations Reflect Meaning Identification for Novel Words in Context

**DOI:** 10.1162/nol_a_00052

**Published:** 2022-02-10

**Authors:** Jacob Pohaku Momsen, Alyson D. Abel

**Affiliations:** Joint Doctoral Program in Language and Communicative Disorders, San Diego State University and UC San Diego, San Diego, CA, USA; School of Speech, Language, and Hearing Sciences, San Diego State University, San Diego, CA, USA

**Keywords:** ERSP, neural oscillations, semantic memory, sentence processing, theta power, beta power

## Abstract

During language processing, people make rapid use of contextual information to promote comprehension of upcoming words. When new words are learned implicitly, information contained in the surrounding context can provide constraints on their possible meaning. In the current study, EEG was recorded as participants listened to a series of three sentences, each containing an identical target pseudoword, with the aim of using contextual information in the surrounding language to identify a meaning representation for the novel word. In half of the trials, sentences were semantically coherent so that participants could develop a single representation for the novel word that fit all contexts. Other trials contained unrelated sentence contexts so that meaning associations were not possible. We observed greater theta band enhancement over the left hemisphere across central and posterior electrodes in response to pseudowords processed across semantically related compared to unrelated contexts. Additionally, relative alpha and beta band suppression was increased prior to pseudoword onset in trials where contextual information more readily promoted pseudoword meaning associations. Under the hypothesis that theta enhancement indexes processing demands during lexical access, the current study provides evidence for selective online memory retrieval for novel words learned implicitly in a spoken context.

## INTRODUCTION

The ability to learn new words and develop an extensive lexicon is a well-studied phenomenon of cognition. Broadly speaking, word learning can be achieved in a number of different ways—via explicit instruction or alternatively through implicit means. When new words are encountered on the fly, meaning has to be inferred from the surrounding linguistic context (Fukkink, 2005; Shtyrov, 2011). For example, if exposed to the unfamiliar word *shap* in the following sentence, *The boy held the shap tightly as he fell asleep*, interpretations would be constrained to potential meanings that are plausibly related to other familiar words in the sentence (e.g., *pillow*, *blanket*). While there is evidence that people can become sensitive to the meaning of a new word after being exposed to it in context only once, long-lasting memory for new words becomes robust after multiple exposures (Horst, 2013).

Learning words from context is often studied using written language (Nagy et al., 1985, 1987), but, importantly, many new words are learned by hearing them used in everyday speech (Carey & Bartlett, 1978; Jenkins et al., 1984; McLeod & McDade, 2011; Saffran et al., 1997). Considering the level of exposure people get to new words over their life span simply by listening to others talk, it is vital to establish an understanding of the brain mechanisms that underpin contextual learning during online speech processing. Extant neuroscientific research on implicit word learning has mostly focused on written text; the present study instead focuses on spoken language processing.

### Neural Oscillations and Speech Processing

An increasingly popular approach to examining EEG data in studies of language processing is using event related spectral perturbations (ERSPs)—a transformation of EEG data that allows for the inspection of both phase and non-phase locked neural activity, which is unobservable using more traditional event-related potential (ERP) analyses (Pfurtscheller & Silva, 1999). ERSPs are formed from a decomposition of the continuous EEG signal to examine how neural activity across various frequency bands changes as a function of different experimental variables. There have been increasing efforts to explain how the neural oscillatory activity works as a vehicle for language-related cognitive operations (see Prystauka & Lewis, 2019).

Both in and out of the context of language processing, theta band activity (∼4–7 Hz) has been most frequently associated with memory retrieval operations (Bastiaansen & Hagoort, 2006; Bastiaansen et al., 2002, 2010; Hald et al., 2006; Schneider & Maguire, 2018). In a 2005 study, researchers found contrasts between the oscillatory response to open-class versus closed-class words during a passive reading task (Bastiaansen et al., 2005). The differences were most stark in theta band activity: Open-class words elicited increases in theta power over left temporal regions of the scalp while comparable modulation was absent for closed-class words. This led to the conclusion that the observed changes were primarily reflective of retrieval processes from long-term memory elicited by semantically rich language. Other studies show theta power enhancement in response to stimuli that are semantically incongruent with preceding language (Bastiaansen & Hagoort, 2006; Bastiaansen et al., 2005; Hald et al., 2006). As for sentence-level dynamics, Lam and colleagues found relative decreases in theta power for words that appeared late in a sentence compared to those that occurred earlier (Lam et al., 2016). Taken together under a functional interpretation, these results suggest that theta power may index effort related to semantic memory retrieval demands, where enhancement during word processing increases if a relevant context does not facilitate lexical access.

In addition, alpha (8–12 Hz) and beta (13–30 Hz) frequencies have been discussed in light of a number of processes likely to play a relevant role during language processing in context, such as memory retrieval, efforts related to controlled attention, predictive processing, and working memory operations (Gao et al., 2017; Hanslmayr et al., 2012; Klimesch, 2012; Klimesch & Schack, 2003; Piai et al., 2014; Weiss & Mueller, 2012). For example, alpha suppression appears to support a range of functions—displaying sensitivity to the complexity of syntactic configurations (Vassileiou et al., 2018), the predictability of upcoming language (Rommers et al., 2017; Wang et al., 2018), and speech intelligibility (Obleser & Weisz, 2012)—and it is generally associated with conditions where cognitive resources are devoted more heavily to the current task (Jensen & Mazaheri, 2010; Klimesch et al., 2007). Studies observing beta oscillations posit a role in actively reflecting the maintenance of context-sensitive meaning representations via top-down predictive signaling (Lewis et al., 2016). This predictive coding account hypothesizes beta band suppression in response to syntactic or semantic perturbations during sentence processing (Bastiaansen et al., 2010; Lewis & Bastiaansen, 2015; Wang et al., 2012). For example, Kielar and colleagues (2014) found that semantic violations in a sentence such as “A new computer will *paint* for many years” (Kielar et al., 2014, p. 3) elicit a decrease in beta and alpha power following the onset of the semantically incongruent word. Together, alpha and beta band suppression may arise in response to increased cognitive demands related to processing words across semantically unrelated contexts—that is to say, contexts that only weakly support contextual learning or inference.

### Neural Oscillations and Word Learning

In addition to assessing learning performance using behavioral measures (i.e., whether a participant can correctly report a new word’s meaning), research has used real-time processing measures to index the brain response to words after a learning opportunity. Previous studies using electrophysiological measures have demonstrated that adults show neural evidence of rapid altered sensitivity to unfamiliar words after being exposed to them in context only once (Batterink & Neville, 2011; Mestres-Missé et al., 2007; Perfetti et al., 2005). While only a few studies have used ERSP measures to compare the processing of unknown relative to known words, theta power appears to exhibit preferential sensitivity to known words especially over the left hemisphere (Krause et al., 2006; Marinkovic et al., 2012), not unlike the greater enhancement seen for semantically rich words over and above closed-class words (Bastiaansen et al., 2005). These studies offer a valuable perspective on the neural oscillations that relate to unfamiliar language.

One notable study provides evidence that theta band activity can act as a useful proxy for some components of the word learning process. In a multisession word learning study, Bakker and colleagues (2015) observed significantly reduced theta band activity when comparing the initial neural response to unfamiliar pseudowords with responses to real words. After a learning session that exposed participants to explicit word definitions, this relative power difference was still present, albeit reduced. When participants were tested on the learned words the following day after an opportunity for overnight memory consolidation, this low frequency response to pseudowords was enhanced such that there was comparable ERSP activity between these newly learned pseudowords and previously known real words. It is currently unknown how oscillatory mechanisms actively support other types of familiarization and learning conditions used to eventually learn words—especially as this occurs in the context of spoken language processing. The current study examines whether effectively using context to build up meaning associations with novel spoken words can instigate a similar transition in the oscillatory response as unfamiliar words eventually trigger online semantic retrieval.

### The Current Study

The ability to attach meaning to unfamiliar words while considering the wider linguistic context is fundamental to successful implicit word learning, and thus warrants a better understanding of the brain mechanisms involved. Thus, the purpose of the current study was to investigate the electrophysiological markers associated with changes in response to novel spoken words in context. This is to say, our aim is not to investigate long-term implicit word learning per se, but instead focuses on one central component of this process: estimating a plausible meaning for a new word based solely on the context it is discovered in. More specifically, we tested the hypothesis that exposure to a novel word embedded across semantically coherent sentences (Meaning (+)) will promote more word-like oscillatory responses than novel words embedded across semantically unrelated sentences (Meaning (−)).

As a supplement to this primary motivation, we also examined unfolding changes in neural activity prior to pseudoword onset to characterize how oscillatory markers of sentence processing and contextual inference correspond to differences in the semantic relatedness across multiple sentences. Participants were exposed to a series of sentence triplets, each containing an identical target pseudoword. Participants were then instructed to identify the meaning of the target pseudoword, requiring them to use adjacent contextual cues embedded across the sentences. In half of the trials, sentence contexts were related to each other so that a representation for the unknown word could fit with all the sentence contexts that the pseudoword had appeared in. Sentence contexts were unrelated in the other half of the trials so that a single meaning could not be associated with pseudowords.

ERSP effects in the theta band typically associated with real-word comprehension provided the primary motivation for our hypothesis. We hypothesized that successfully associating meaning to pseudowords would coincide with increased theta power enhancement to these words between their first and final presentation, and that a similar increase would be diminished or not occur to pseudowords lacking a meaning association. We also expected alpha and beta suppression to disproportionately occur as participants processed words in unrelated contexts, that is to say when expectations about upcoming words are more frequently violated (Kielar et al., 2014).

## METHODS

### Participants

The current study included a cohort of 32 monolingual English-speaking, right-handed adult participants. All adults were administered a nonword repetition task (Dollaghan & Campbell, 1998), which served as an index of phonological working memory ability (percentage of consonants correct *M* = 94.0%; *SD* = 4.31%). Data from four participants were removed from the final analysis due to excessive artifacts during recording, resulting in a final sample of 28 participants (*M*_age_ = 20.5, *SD*_age_ = 2.17; all female). Participants gave informed consent in accordance with the San Diego State University Institutional Review Board. All participants gave informed consent for their participation and were compensated with academic course credit.

### Stimulus Materials

Our paradigm used a total of 300 spoken sentence stimuli in which the terminal word was always a noun generated from a database of nouns commonly acquired by the age of 30 months (MacArthur-Bates Communicative Developmental Inventory; Fenson et al., 2006). Sentences were all 6–9 words in length, and the terminal noun was preceded by either a determiner (*a*, *the*) or a possessive (*my*, *your*, *his*/*her*).

For the word learning paradigm, trial stimuli were created by combining sentences into sets of three (described in more detail below). The sentence-terminal noun in all three sentences within a given trial was replaced by the same pseudoword, generated from a database (Storkel, 2013) of consonant-vowel-consonant sequences. None of the pseudowords had word-initial sounds of /s/ or /sh/ to improve the time-locking accuracy to the pseudoword. Additionally, there were no phonetic constraints for vowels or word-final consonants.

All sentence stimuli were normed in an offline sentence completion task given to a cohort of 248 adults to produce cloze probability ratings for each sentence-final word. The cloze probability of a word is defined as the percentage of people who spontaneously choose to finish a sentence with that word in an offline norming task. Cloze probability information from the norming task was used to contrive two conditions. The Meaning (+) condition incrementally facilitated learning for the target pseudoword within a trial by increasing the amount of contextual support for each sentence. This was achieved by using sentences whose final noun had low cloze probabilities in the offline norming task as the first out of three sentences in a trial, and high cloze probabilities as the trial-final sentences (Mean cloze probabilities for target words in first Meaning (+) sentences = 4.0%, *SD* = 6.0%; in second Meaning (+) sentences = 40.6%, *SD* = 10.0%; in third Meaning (+) sentences = 88.1%, *SD* = 11.2%).

Importantly, the Meaning (+) condition used sentences from the norming task with the same final real word within each trial, making the sentences complimentary in terms of their support for one target meaning. In the Meaning (−) condition, the pseudowords also replaced the sentence-final word in each sentence stimuli; however, the real word that the pseudoword replaced was a different real word for each sentence in the triplet (see Table 1) (Mean cloze probabilities for target words in first Meaning (−) sentences = 17.5%, *SD* = 17.7%; in second Meaning (−) sentences = 20.3%, *SD* = 20.2%; in third Meaning (−) sentences = 17.1%, *SD* = 15.6%).

**Table 1.** Example triplets from each condition

**Meaning (+) condition**		
**Sentence order**	**Sentence presented**	**Real word replaced**
1	Her parents bought her a ***pav***	bed
2	The sick child spent the day in his ***pav***	bed
3	Mom piled the pillows on the ***pav***	bed
**Meaning (−) condition**		
**Sentence order**	**Sentence presented**	**Real word replaced**
1	Don’t drop and break the ***pav***	cup
2	I spilled dinner all over my ***pav***	pants
3	You have to study hard to be a ***pav***	doctor

Each participant was administered 100 trials (sentence triplets), 50 Meaning (+) trials and 50 Meaning (−) trials. Confounds introduced from by-item effects were controlled for by using each pseudoword stimuli in both the Meaning (+) and Meaning (−) conditions across participants. All sentences were recorded in a sound-attenuated chamber by a female native English speaker, and were produced at a natural pace to promote naturalistic listening conditions (Mean sentence length prior to pseudoword Meaning (+) = 1,788.5 ms, *SD* = 342.0 ms; Meaning (−) = 1,883.4 ms, *SD* = 347.4 ms; Mean pseudoword length Meaning (+) = 544 ms, *SD* = 81 ms; Meaning (−) = 534 ms; *SD* = 78 ms).

### Procedure

The experimental paradigm was adopted from a 2007 study investigating implicit word learning in adults (Mestres-Missé, et al., 2007). Participants listened to sets of three naturally-paced sentences. Participants were instructed to attend to the three sentences and to attempt to identify the meaning of the unknown pseudoword by using contextual information provided across the sentence triplet. At the beginning of a trial, a fixation cross appeared on a monitor screen for 600 ms. After this fixation cross, a spoken sentence stimulus was presented through speakers arranged approximately 1 meter from the participant. After the offset of the first sentence, participants saw a screen with multiple fixation crosses indicating that they could push a button to initiate the presentation of the next sentence. After a button push to progress the trial, another fixation cross appeared on the screen for 600 ms prior to the onset of the next sentence to ensure that each sentence onset had a baseline period without stimulus presentation. After the third sentence in a trial was presented, participants gave a button push to initiate the response phase of the trial, in which they were asked by an experimenter if they believed the target pseudoword was meaningful (i.e., if a single word was semantically congruent with the entire sentence triplet). Importantly, these questions were not verbalized until after participants ended the trial with a self-paced button push. If the participant believed the target pseudoword was meaningful, they gave an oral response to the experimenter in an attempt to identify the specific word that would fit best to replace the pseudoword in that particular trial.

**Figure 1.**
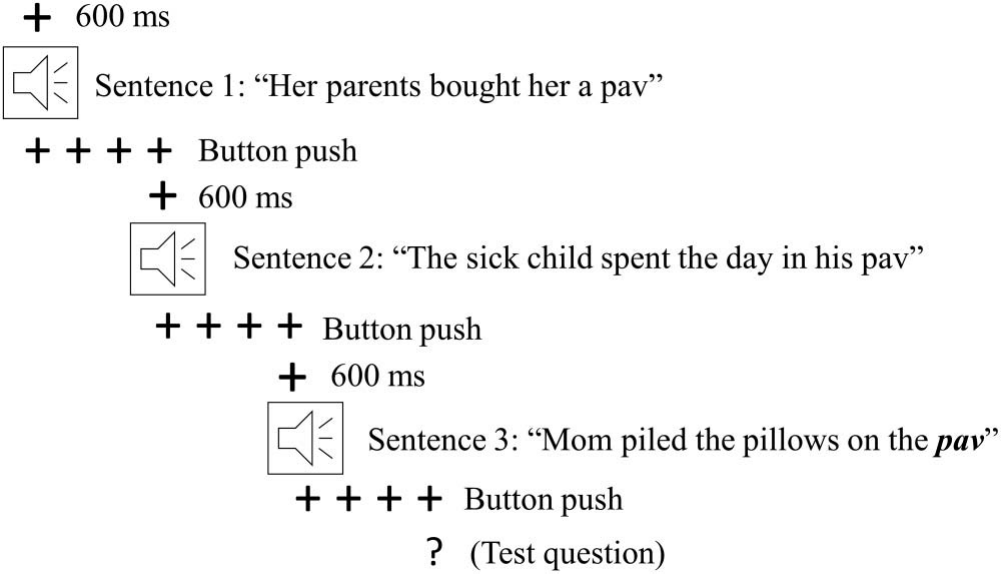
Example of one Meaning (+) trial.

Trials in the Meaning (+) condition were only counted as correct if the participant successfully identified the target word that the pseudoword replaced or gave an answer that was reasonably similar (e.g., a response “*truck*” for the target “*car*”). Trials in the Meaning (−) condition were counted as correct if the participant reported that the target pseudoword could not represent a noun that was semantically congruent across all three sentences within the trial (e.g., “*the word is not meaningful*”). Only correct trials were used in the EEG analysis. Prior to testing, a training session was administered, such that each participant was exposed to an example trial in the Meaning (+) and Meaning (−) conditions. Feedback was given for performance during the training session but not for the actual experiment. See Figure 1 for a visual representation of a single trial.

### EEG Acquisition and Processing

EEG recording was performed with a 64 electrode Neuroscan Quickcap (compumedicsneuroscan.com/) arranged according to the 10–20 International Standard configuration system. The EEG signal was referenced online at a central electrode site near the vertex of the head and rereferenced offline to the average of two lateral electrode sites approximate to the mastoids (CB1, CB2). During recording, EEG signals were low-pass filtered (200 Hz) and sampled at 1 kHz. Data were later resampled offline at 512 Hz. A 40 Hz low-pass and 1 Hz high-pass filter were applied to the data and then independent component analysis was performed on the continuous EEG recording for each subject in order to identify sources of nonbrain-related electrical activity (Infomax ICA; Bell & Sejnowski, 1995). Components identified as being primarily related to blink and movement artifacts were subsequently removed after manual inspection (Mean components rejected = 1.77; *SD* = 0.91). Malfunctioning electrodes were corrected by spherical interpolation based on adjacent electrode data. Any remaining artifacts related to eye movements and flatlining were flagged with an automatic artifact detection algorithm (EEGLAB; Delorme & Makeig, 2004) and removed from analysis. Additionally, epochs corresponding to trials with an incorrect response from the behavioral task were removed from the analysis. (*M* (*SD*) number of trials per subject entering into the analyses: Meaning (+) 1st sentence = 36.4 (4.1); Meaning (+) 2nd sentence = 37.6 (4.3); Meaning (+) 3rd sentence = 37.8 (4.2); Meaning (−) 1st sentence = 39.1 (3.9); Meaning (−) 2nd sentence = 39.9 (3.8); Meaning (−) 3rd sentence = 40.8 (3.9).

### Time–Frequency Analysis

Statistical analyses of time–frequency data used nonparametric cluster-based permutation tests (Maris & Oostenveld, 2007). This approach is particularly appropriate for analyses in which there is less certainty regarding the nature and time course of the effects of interest. For conditional comparisons of interest, a *t* value was derived from each data point across two subject-channel-frequency-time matrices. A three-dimensional cluster statistic was derived from summed *t* values adjacent in location, time, and frequency (alpha cluster threshold = 0.025). Cluster-level statistics were compared with a null distribution of cluster statistics obtained via a randomized permutation procedure (*N* = 1,000). Monte Carlo *p* values were acquired by comparing the observed cluster statistic to this distribution. Electrode neighbors were defined via triangulation method (6.3 average neighbors per electrode). Time–frequency representations were computed via short-time fast Fourier transformation using Fieldtrip code implemented in MATLAB (ver. R2019b) (mathworks.com; Oostenveld et al., 2011). A 500 ms sliding Hann window was applied to epochs using 32 ms time steps and across frequencies from 2–30 Hz at steps of 2 Hz.

Data segments used in the cluster analyses were generated using epochs that were time-locked to the onset of the critical pseudoword in each sentence. These epochs included data spanning from 1,000 ms prior to and 750 ms after pseudoword onset. To help delineate if effects were specific to pseudoword processing rather than their preceding context, we performed similar but separate analyses of target data recorded prior to (−1,000 to 0 ms) and after (0 ms to 750 ms) the pseudoword onset in each sentence. To observe differences in pseudoword processing relative to task-related changes in brain activity that accumulate within trials, pre-sentence baselines were used to normalize target activity related to pseudowords. Separate epochs time-locked to sentence onset were extracted from continuous data to generate time–frequency epochs for baseline corrections. Data from 250 ms to 50 ms prior to sentence onset was averaged across Meaning (+) and (−) conditions for each sentence presentation, resulting in three time–frequency baseline arrays used for point-by-point decibel transformation (10*log10(activity/baseline)) of pseudoword related activity to create ERSPs for all reported statistical analyses and visual representations of the data.

We investigated the neural correlates related to novel word processing in context by analyzing the neural response to pseudowords embedded across either semantically coherent or incoherent sentences. To test whether being associated with a meaning representation changes the trajectory of the neural response to pseudowords, we tested an interaction between Meaning and sentence presentation, that is, a comparison between the Meaning (+) and (−) conditions for the difference between the power at pseudoword onset in the first and final sentence. Furthermore, we examined direct comparisons of pre-pseudoword activity at each sentence presentation (sentences 1, 2, and 3) to identify how our Meaning manipulation modulated online sentence processing dynamics as participants attempted to use contexts to inform novel word processing.

## RESULTS

### Behavioral Performance

Trial accuracy across both the Meaning (+) and Meaning (−) conditions were relatively high. Participants correctly identified a meaning for the target pseudoword in the Meaning (+) condition on 83.8% (*SD* = 7.9%) of trials. Participants also correctly reported that the pseudoword did not represent a plausible word across sentence contexts in the Meaning (−) condition on 91.0% (*SD* = 5.6%) of trials. A paired *t* test suggested that task performance was slightly enhanced in the Meaning (−) condition (*t* = 3.9; *p* < 0.01). Overall high performance suggests that the memory and learning demands posed by the current task were relatively manageable for our cohort of healthy adults.

### Cluster-Based Permutation Results

Cluster-based permutation tests comparing the difference in pseudoword processing from initial to final presentation across Meaning (+) and (−) trials indicated rejection of the null hypothesis of exchangeability across data distributions (*p* = 0.049). This nonparametric analysis generated a cluster estimate in the positive direction that included low frequency estimates (2–4 Hz) and lasted from approximately 500 ms post-pseudoword onset to the end of the epoch. This result suggests that the pseudowords that eventually triggered semantic retrieval by being positioned in meaningful contexts elicited greater increases of low-frequency power in the theta band compared to pseudowords embedded in contexts that precluded a similar semantic representation from being retrieved. Similar cluster-based tests directly comparing pre-pseudoword activity across Meaning (+) and (−) trials did not identify significant differences for first or second sentences. A test comparing Meaning (+) and (−) for the third sentences indicated a significant difference, revealing a cluster in the negative direction (*p* = 0.002). This test was associated with a cluster estimate predominantly characterized by frequency values in the alpha and low beta range (8–20 Hz), which began near the initial time sample and lasted until approximately 300 ms prior to pseudoword onset.

### Theta Power Enhancement Across Pseudoword Presentation

**Figure 2.**
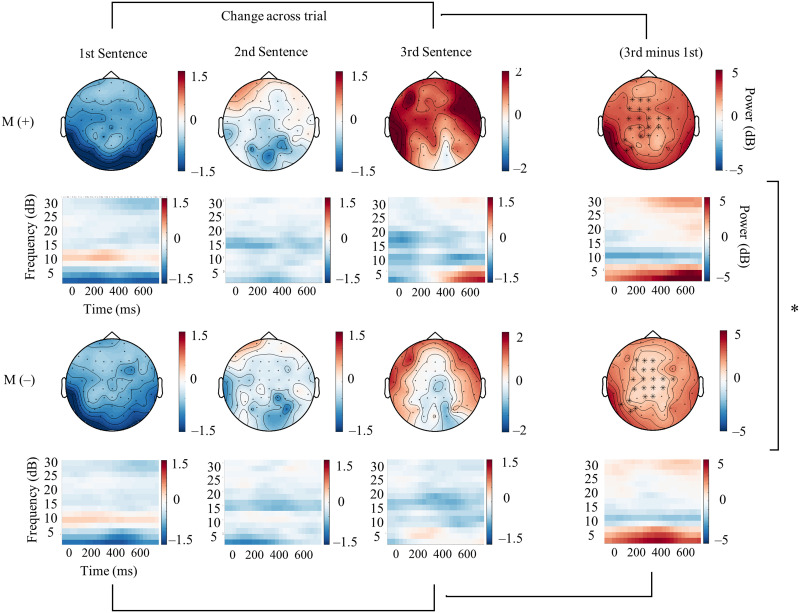
A series of topographical distributions and time–frequency plots representing the change in theta band power occurring after pseudoword onset relative to the pre-sentence baseline. An interaction between Sentence presentation (transition between 1st and 3rd sentences) and the Meaning manipulation (Meaning (+) vs. (–) trials) revealed greater changes in theta enhancement for pseudowords associated with meaning (far right). The shown topographical distributions of theta enhancement correspond to the spatiotemporal features of the cluster estimate revealed by this interaction (2–4 Hz; approximately 500–750 ms post-pseudoword onset). The time–frequency plots display ERSP activity averaged across all channels identified in the interaction, which are indicated by the asterisks on the far-right scalp plots.

**Figure 3.**
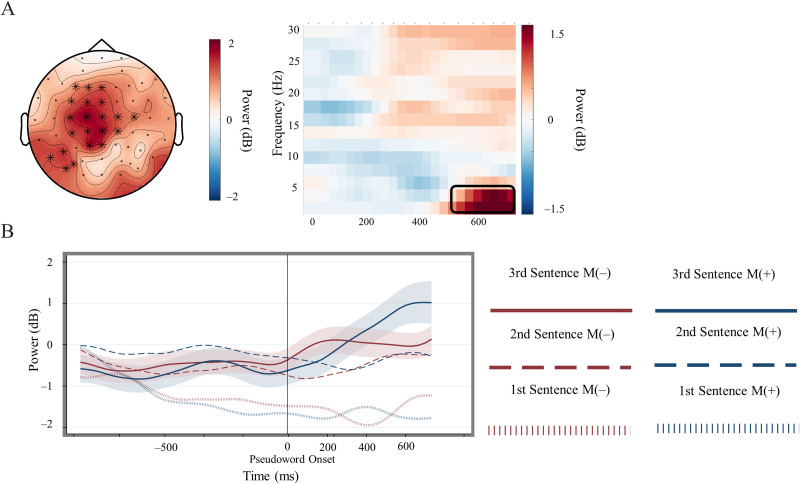
(A) Topographical distribution and time–frequency plot depicting the difference in theta enhancement observed across Meaning (+) relative to pre-sentence baseline relative to Meaning (−) trials. The scalp map shows averaged theta power over from approximately 500 ms post-pseudoword onset to the end of the epoch. The spectrogram includes ERSP data averaged across all channels indicated by asterisks in the scalp plot. (B) Time course of theta power averaged within spatiotemporal cluster boundaries for each sentence across Meaning conditions. Shading for third sentence data indicates ±1 standard error of the mean and illustrates that theta power related to pseudowords completing Meaning (+) trials was uniquely enhanced relative to pre-sentence baseline. 0 ms corresponds to pseudoword onset.

Compared to the difference between pseudowords upon their initial presentation, final pseudowords embedded within semantically cohesive sentence sets and consequently associated with meaning elicited greater theta enhancement compared to pseudowords that appeared across a series of mutually unsupportive sentences (Figure 2). Our nonparametric analysis suggested that this effect manifested relatively late, from approximately 500 ms after pseudoword onset until the end of the epoch (see Sassenhagen & Draschkow, 2019, for discussion about the interpretive limitations of cluster-based tests). The twenty-one electrodes comprising this cluster estimate were predominately over central electrode locations, but also included electrodes over left posterior scalp regions (Figure 3). Paired-sample *t* tests using subject-averaged data from third sentences indicated that theta power in Meaning (+) trials was greater than zero, indicating a relative power enhancement compared to the pre-sentence baseline (*t* = 2.34, *SD* = 2.14, *p* < 0.05). Alternatively, the theta response to third pseudowords in Meaning (−) trials was not significantly enhanced compared to the baseline (*t* = 0.05; *p* = 0.9).

### Relative Alpha and Beta Power Suppression Prior to Final Pseudowords

**Figure 4.**
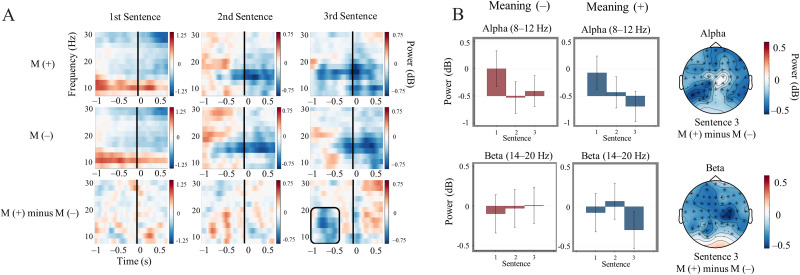
(A) Spectrograms including data averaged across significant cluster between third Meaning (+) and (−) sentences. Time and frequency range identified by the significant cluster are highlighted by the box in the third sentence difference plot (right side, bottom row). (B) Topographical distribution of the difference in alpha and beta activity across Meaning conditions prior to pseudoword onset reflected in the cluster analysis (−950 to −330 ms in the figure). Bar plots on left display alpha and beta activity corresponding to the observed cluster estimate across sentences. Lines in these plots provide a time stamp of pseudoword onset.

Our analyses indicated relative alpha and beta band (8–20 Hz) suppression preceding final pseudoword onset in Meaning (+) trials relative to Meaning (−). The separate calculation of group means for alpha (8–12 Hz) and beta (14–20 Hz) band activity suggested that differences prior to pseudoword onset were driven predominately by beta band suppression in the Meaning (+) condition (*t* = −2.79, *SD* = 0.56, *p* < 0.01; Figure 4B). Electrodes in the significant cluster indicated a widely distributed effect unlike the relative left-lateralization seen for the theta band enhancement.

## DISCUSSION

The current study set out to explore oscillatory neural dynamics that support implicit word learning from speech. We tested the hypothesis that adults can develop an online sensitivity to the meaning of unfamiliar words embedded across meaningful spoken sentence contexts by analyzing oscillatory signatures of lexical processing. We hypothesized that greater theta power enhancement would occur in association with pseudowords after being embedded across semantically coherent as opposed to unrelated sentences. Our data revealed that pseudowords eliciting a meaning representation were associated with greater theta power enhancement across trials relative to pseudowords presented in contexts where meaning was not similarly activated. Additionally, we observed more alpha and beta power suppression prior to final pseudowords in Meaning (+) trials relative to Meaning (−).

### Pseudowords Associated with Meaning Elicit Theta Band Enhancement

When novel or unfamiliar words are processed for the first time, low frequency activity in the ERSP response lacks features common to real-word comprehension (Bakker et al., 2015; Marinkovic et al., 2012). The present study found that processing novel spoken words in context can engage oscillatory mechanisms related to real-word processing soon after their initial presentation. While similar effects had been demonstrated in response to written text (e.g., Mestres-Missé et al., 2007), the current study provides a novel contribution by examining this processing in the context of spoken language. This finding is significant, especially considering the importance of word learning from speech. Importantly, we found that these effects depended on the association between novel words and meaning representations—a direct product of the context that pseudowords were presented in. A “meaningful” pseudoword embedded across semantically coherent sentences eventually elicited greater theta band enhancement on average than pseudowords embedded across incoherent contexts where meaning identification was not achieved. This activity is unlikely the result of pseudoword repetition across sentences; indeed, the effect displayed spatiotemporal features similar to those found in previous studies investigating ERSP correlates of language processing that suggest this low frequency enhancement is linked to lexical retrieval processes (Bastiaansen et al., 2002, 2005; Hald et al., 2006; Kielar et al., 2014).

Other neuroimaging work on incidental word learning in the written modality has provided evidence of canonical language network activation (e.g., left middle temporal and inferior frontal gyri) in conditions where novel words were meaningful in their provided context but also in cases where context did not support meaning identification (Mestres-Missé et al., 2008). A 2012 study used high density MEG and EEG to estimate source generators for theta effects during a lexical decision task using real and pseudowords. Researchers observed more theta band activation in left temporal and left inferior frontal regions for real words than pseudowords between approximately 400–500 ms post-word-onset (Marinkovic et al., 2012). More recent work established a similar generator for pseudoword related activity after a word learning opportunity (Bakker-Marshall et al., 2018). In line with this work, the current effect exhibited a lateralized bias and extended over left posterior temporal areas, reminiscent of the left-biased topography common to EEG studies of lexical-semantic retrieval (Bastiaansen et al., 2005; Marinkovic et al., 2012; Salisbury & Taylor, 2012).

Prior work also demonstrated activity across several other sites in the left hemisphere that was unique to processing new words across meaningful relative to non-meaningful contexts, including anterior parahippocampal gyrus, thalamus, and precuneus (Mestres-Missé et al., 2008). There is growing support for a role of the medial temporal lobe during online language processing—not only for learning but also when ambiguities about word meaning need to be resolved (Duff & Brown-Schmidt, 2012; Pu et al., 2020). Links between theta rhythms and functional coordination involving the hippocampus during memory retrieval are also consistent with the observed effects in the Meaning (+) trials, considering that meaning identification in the current paradigm requires a sensitivity to information delivered in prior contexts (Herweg et al., 2016).

Previous evidence tracking the emergence of theta enhancement to unknown words after meaning acquisition was demonstrated using explicit learning opportunities, and this effect of learning was heightened after a period of overnight consolidation (Bakker et al., 2015; Bakker-Marshall et al., 2018). The present study extends previous work by demonstrating that a similar effect can be achieved via implicit learning mechanisms: As adults used sentence contexts to successfully associate meaning with novel word forms, theta band activation was enhanced by the third time that pseudowords were heard. Importantly, because we did not test participants on their memory for pseudoword-meaning associations after the experiment, we cannot make claims about the extent to which new words were learned—indeed, the task was a meaning identification task not a word learning task per se. The importance of our findings lies in the discovery that people can quickly develop an online sensitivity to the meaning of unfamiliar words after hearing someone use them appropriately—words elicited an online neural signature suggestive of meaning acquisition from context only when words appeared in the Meaning (+) condition.

An important caveat to our findings involves the role of contextual expectations during sentence or discourse level processing. Our effect may be similar to that observed by Rommers and colleagues (2017), who crossed effects of sentence constraint and word predictability and found that target-word theta band enhancement was most strongly elicited by unexpected words when participants were better able to generate expectations for them (i.e., when completing high constraint sentences). Because our goal was to experimentally contrive success rates for implicit pseudoword-meaning associations, target real words completing Meaning (+) trials did have higher cloze-probabilities on average than target words in Meaning (−) trials. Thus, a current limitation of this study is that we cannot determine how much of this effect is a product of these differences in context-driven expectations and their subsequent violation. Indeed, this leaves the door open for an alternative interpretation: The effect could be taken to reflect increased information processing demands as a result of competition between different lexical representations partially activated by the end of Meaning (+) trials. If Meaning (+) trials drove participants to activate one or multiple related candidates for the meaning identification task, competition among different meaning representations or even between retrieved lexical entries and the novel word itself could potentially explain increases in theta band activity. For example, using a single-word auditory lexical decision task, Strauß et al. (2014) observed more theta enhancement in response to phonologically ambiguous pseudowords (e.g., “banene”) compared to real words (*banana*) or other pseudowords with novel phonological constructions. Meaning associations unique to Meaning (+) trials may have had a similar effect by resulting in co-activation between lexical representations in long-term memory and unfamiliar wordforms. Thus, theta enhancement may index a conflict resolution process elicited by activated competing lexical representations.

Lastly, theta activity after final pseudowords in Meaning (−) trials corresponding to the spatiotemporal estimates of the cluster interaction was not significantly different from zero, suggesting that a similar effect was not generated when pseudowords remained “meaningless.” However, upon inspecting the transition from initial to final pseudowords in this condition, we found that these stimuli do exhibit relatively increased theta power compared to pre-sentence baseline across presentations; and a post hoc cluster-permutation test directly comparing first and third pseudowords in these trials confirms this observation (*p* < 0.001). We find it feasible that low frequency activity tied to working memory operations may account for differences as the trials progress. Maintaining relevant information from earlier sentences within the same trial is presumably important for task performance, and memory demands during Meaning (−) trials may even be heightened due to the semantic incongruence between sentences. Theta activity reflecting more general working-memory encoding processes would align it with studies that observed theta power enhancement during successful memory performance, especially when working memory content involved sequential information (Axmacher et al., 2006; Kleberg et al., 2014; Klimesch et al., 2001; Nyhus & Curran, 2010; Roux & Uhlhaas, 2014; Scholz et al., 2017). Because we used naturally paced speech and because our task required that all trials involve a correct assessment of contextual fit, we believe it is possible that working-memory-related theta activity could contribute to ERSP activity in either Meaning condition.

### Alpha and Beta Suppression Within Meaningful Contexts

In addition to the observed effects in the theta band, beta and alpha suppression was relatively heightened prior to final pseudoword onset in Meaning (+) trials compared to Meaning (−). Across different sensorimotor domains, a number of proposals have posited beta power as an index reflecting the preparation for processing anticipated events—inversely related to the expected probability of voluntary action execution or of stimulus presentation (Jenkinson & Brown, 2011; Van Ede et al., 2011). Applied to language processing, interplay between top-down predictions, instantiated by beta activity, and bottom-up error signaling might play a role in maintaining and updating a representation of discourse context important for flexible online comprehension (Lewis et al., 2016; Meyer, 2018). Within this framework, beta band suppression may have occurred selectively as people updated relevant information about trial content or pseudoword meaning, whereas in Meaning (−) trials the semantic incoherence across sentences prevented stable contextual representations from being formed to begin with—which is likely the reason we did not find evidence for similar suppression effects in response to the pseudowords themselves

Additionally, recent work using MEG directly tested the relationship between power suppression effects during sentence encoding and word predictability, and found that neither alpha nor beta band activity held a monotonic relationship with the level of contextual constraint provided by the sentence context (Terporten et al., 2019). This indicates these mechanisms are not merely an index of upcoming word predictability and may instead relate more closely to information updating during sentence processing. We believe a compatible description can be framed around general long-term memory encoding and retrieval, in which both alpha and beta activity act as an important mechanism for long-term memory access (Hanslmayr et al., 2012; Klimesch, 2012; Klimesch et al., 2005). For example, alpha and beta suppression can increase as a function of the number of items retrieved from memory (Khader & Rösler, 2011), and more relevantly, low beta suppression has been observed in response to real words compared to unknown novel words (Bakker et al., 2015). Together, the fact that Meaning (+) trials saw disproportionately enhanced beta and alpha suppression may reflect more robust semantic activation or updating compared to Meaning (−).

Other potential interpretations could be mounted from evidence showing beta power as closely related to reward processing (Lansink et al., 2016; Schwerdt et al., 2020). This may be relevant to our current findings, considering that previous work has shown that subcortical nuclei important for reward processing can be engaged during implicit language learning even in the absence of explicit feedback about learning performance (Ripollés et al., 2014, 2016). Lastly, it is also possible these effects were in part driven by differences in general attentional engagement during these sentences (Jensen & Mazaheri, 2010; Klimesch et al., 2007; Shahin et al., 2009).

### Conclusions

We presented data from one of the first studies to focus on oscillatory brain dynamics related to processes important for implicit word learning from speech. In summary, a cohort of 28 healthy adults were able to integrate contextual information across a series of three sentences in order to estimate a viable association between a novel spoken word and a meaningful concept. Sensitivity to context was reflected by a greater progressive enhancement of theta band activity in trials with meaningful pseudowords compared to trials where pseudowords remained meaningless. This work helps make progress toward understanding how people learn from hearing words used appropriately by other speakers by showing that the brain adapts quickly to speech to support the application of meaning associations to previously unknown word forms.

## ACKNOWLEDGMENTS

The authors would like to acknowledge Julie Schneider for her feedback on this manuscript, members of the Language Learning Lab at San Diego State University for their assistance with data collection and processing, and the research participants. Jacob Pohaku Momsen was supported by the Tribal Membership Initiative Fellowship through UC San Diego. Alyson D. Abel was supported by the National Institute of Deafness and other Communication Disorders of the National Institutes of Health under award number R21 DC018865, the San Diego State University Grants Program, and the National Science Foundation under award number BCS-1551770.

## FUNDING INFORMATION

Alyson Abel, National Science Foundation (https://dx.doi.org/10.13039/100000001), Award ID: Grant BCS-1551770. Alyson Abel, National Institute of Deafness and other Communication Disorders of the National Institutes of Health (https://dx.doi.org/10.13039/100000055), Award ID: R21 DC018865.

## AUTHOR CONTRIBUTIONS

**Jacob Pohaku Momsen**: Methodology; Data curation; Formal analysis; Writing—review & editing. **Alyson D. Abel**: Conceptualization; Data curation; Methodology; Writing—review & editing; Funding acquisition; Supervision.

## TECHNICAL TERMS

Neural oscillations: In the current study, this refers to large-scale repetitive and synchronized neural activity that can be measured with scalp-recorded EEG.
Phonological: Refers to the system of speech sounds that constitute words.
Cloze probability: The probability that a particular word will appear in a target sentence position given its prior context.
Sentence constraint: Related to the highest cloze probability that a sentence affords.
Monotonic: Describes a function that only entirely increases or decreases in value.
